# Supplementary material for: Heart rate and age modulate retinal pulsatile patterns
Source: Commun Biol. 2022 Jun 14;5:582. doi: 10.1038/s42003-022-03441-6 (PMC9197857; doi:10.1038/s42003-022-03441-6)
Supplement: Supplementary file 2 — Reporting Summary [file 42003_2022_3441_MOESM2_ESM.pdf]

nature portfolio

Corresponding author(s): DBPR COMMSBIO-21-2626

Last updated by author(s): Apr 19, 2022

Reporting Summary

Nature Portfolio wishes to improve the reproducibility of the work that we publish. This form provides structure for consistency and transparency in reporting. For further information on Nature Portfolio policies, see our Editorial Policies and the Editorial Policy Checklist.

Statistics

For all statistical analyses, confirm that the following items are present in the figure legend, table legend, main text, or Methods section.

n/a	Confirmed	
☐	☒	The exact sample size (*n*) for each experimental group/condition, given as a discrete number and unit of measurement
☐	☒	A statement on whether measurements were taken from distinct samples or whether the same sample was measured repeatedly
☐	☒	The statistical test(s) used AND whether they are one- or two-sided<br>*Only common tests should be described solely by name; describe more complex techniques in the Methods section.*
☐	☒	A description of all covariates tested
☐	☒	A description of any assumptions or corrections, such as tests of normality and adjustment for multiple comparisons
☐	☒	A full description of the statistical parameters including central tendency (e.g. means) or other basic estimates (e.g. regression coefficient) AND variation (e.g. standard deviation) or associated estimates of uncertainty (e.g. confidence intervals)
☐	☒	For null hypothesis testing, the test statistic (e.g. *F*, *t*, *r*) with confidence intervals, effect sizes, degrees of freedom and *P* value noted<br>*Give P values as exact values whenever suitable.*
☒	☐	For Bayesian analysis, information on the choice of priors and Markov chain Monte Carlo settings
☒	☐	For hierarchical and complex designs, identification of the appropriate level for tests and full reporting of outcomes
☐	☒	Estimates of effect sizes (e.g. Cohen's *d*, Pearson's *r*), indicating how they were calculated

Our web collection on statistics for biologists contains articles on many of the points above.

Software and code

Policy information about availability of computer code

Data collection	uEye Cockpit (iDS, Germany)
Data analysis	The MATLAB R2017b programming environment (MathWorks, Natick, USA) with academic license.<br>The open-source Retina Imaging Toolbox (https://github.com/ivanalabounkova/retinaimagingtoolbox; GNU GPL version 3 license).

For manuscripts utilizing custom algorithms or software that are central to the research but not yet described in published literature, software must be made available to editors and reviewers. We strongly encourage code deposition in a community repository (e.g. GitHub). See the Nature Portfolio guidelines for submitting code & software for further information.

Data

Policy information about availability of data

All manuscripts must include a data availability statement. This statement should provide the following information, where applicable:

- Accession codes, unique identifiers, or web links for publicly available datasets
- A description of any restrictions on data availability
- For clinical datasets or third party data, please ensure that the statement adheres to our policy

De-identified data sets can be made available upon a reasonable email request to Ivana Labounkova (ilabounk@umn.edu), Dr. Horn K. Folkert (folkert.horn@augen.imed.uni-erlangen.de), or another responsible personnel from the Department of Ophthalmology and University Eye Hospital, Friedrich-Alexander University Erlangen-Nürnberg at Erlangen, Erlangen, Germany.

Field-specific reporting

Please select the one below that is the best fit for your research. If you are not sure, read the appropriate sections before making your selection.

☒ Life sciences ☐ Behavioural & social sciences ☐ Ecological, evolutionary & environmental sciences

For a reference copy of the document with all sections, see nature.com/documents/nr-reporting-summary-flat.pdf

Life sciences study design

All studies must disclose on these points even when the disclosure is negative.

Sample size	The sample size was designated by the Erlangen Glaucoma registry, which was prospectively collecting data of healthy subjects and patients with various eye conditions. The data of the participants who underwent the acquisition of video-ophthalmoscopic data (i.e., monocular or binocular retinal video-recordings - RVR) formed two cohorts in the current study (i.e., healthy participants and participants with diagnosed ocular hypertension - OHT subjects). These two cohorts consist of non-glaucomatic subjects with normal IOP at the time of data collection. Thus, we used all the available data of healthy and OHT subjects who had the RVR. No previous sample-size calculation was performed as the findings of the study were novel and hitherto suggested in theoretical models without any numerical outcomes.<br>The dataset includes balanced numbers in each group with even distribution of men and women.
Data exclusions	No data were excluded from the analysis.
Replication	In a total of 34 retinal video-recordings (RVRs), used principal component analysis detected spontaneous venous pulsation pattern in 33 RVRs (97%) and optic cup pulsation pattern in 31 RVRs (91%). The principal component analysis failed due to a low video data quality.
Randomization	Data provided by Erlangen glaucoma registry includes the classification of the subjects according to their physical condition evaluated by physicians in University Eye Hospital, Friedrich-Alexander University Erlangen- Nurnberg. No further group allocation was performed.
Blinding	Blinding was not relevant to the study.

Reporting for specific materials, systems and methods

We require information from authors about some types of materials, experimental systems and methods used in many studies. Here, indicate whether each material, system or method listed is relevant to your study. If you are not sure if a list item applies to your research, read the appropriate section before selecting a response.

Materials & experimental systems

n/a	Involved in the study	
☒	☐	Antibodies
☒	☐	Eukaryotic cell lines
☒	☐	Palaeontology and archaeology
☒	☐	Animals and other organisms
☐	☒	Human research participants
☐	☒	Clinical data
☒	☐	Dual use research of concern

Methods

n/a	Involved in the study	
☒	☐	ChIP-seq
☒	☐	Flow cytometry
☒	☐	MRI-based neuroimaging

Human research participants

Policy information about studies involving human research participants

Population characteristics	14 healthy controls (8 females), age 66.0+-13.2 years old<br>16 ocular hypertension patients with pharmacologically normalized intraocular pressure (7 females), age 58.7+-12.9<br>All values of intraocular pressure and retinal nerve fiber layer (RNFL) were in physiological ranges.<br>Eye examination values (refractive error, visual acuity, perimetry) were within physiological ranges without any significant pathology.
Recruitment	The data of the participants who underwent the acquisition of video-ophthalmoscopic data (i.e., monocular or binocular retinal video-recordings - RVR) formed two cohorts in the current study (i.e., healthy participants and participants with diagnosed ocular hypertension -OHT subjects). These two cohorts consist of non-glaucomatic subjects with normal IOP at the time of data collection. Thus, we used all the available data of healthy and OHT subjects who had the RVR.
Ethics oversight	Ethical committee at the Friedrich-Alexander University of Erlangen-Nürnberg

Note that full information on the approval of the study protocol must also be provided in the manuscript.

Clinical data

Policy information about clinical studies

All manuscripts should comply with the ICMJE guidelines for publication of clinical research and a completed CONSORT checklist must be included with all submissions.

Clinical trial registration	NCT00494923
Study protocol	https://clinicaltrials.gov/ct2/show/NCT00494923?term=NCT00494923&draw=2&rank=1
Data collection	The monocular and binocular video-ophthalmoscopic video-recording were acquired between January 2015 and December 2017 at Fridrich-Alexander University of Erlangen-Nurnberg, Germany
Outcomes	Primary outcome measure was Pearson correlation coefficient between heart rate, age and morphological measurements of retinal pulsatile patterns. Secondary outcome measure was impact of heart rate and age as covariates in between-group difference testings. p-values or change in p-values assessed the outcome measures.
